# Uncovering MicroRNA Regulatory Hubs that Modulate Plasma Cell Differentiation

**DOI:** 10.1038/srep17957

**Published:** 2015-12-11

**Authors:** Dong-Yan Tsai, Kuo-Hsuan Hung, I-Ying Lin, Shin-Tang Su, Shih-Ying Wu, Cheng-Han Chung, Tong-Cheng Wang, Wen-Hsiung Li, Arthur Chun-Chieh Shih, Kuo-I Lin

**Affiliations:** 1 Genomics Research Center, Academia Sinica, Taipei 115, Taiwan; 2 Institute of Biochemistry and Molecular Biology, National Yang-Ming University, Taipei 112, Taiwan; 3 Institute of Microbiology and Immunology, National Yang-Ming University, Taipei 112, Taiwan; 4 Institute of Information Science, Academia Sinica, Taipei 115, Taiwan; 5 Biodiversity Research Center, Academia Sinica, Taipei 115, Taiwan

## Abstract

Using genome-wide approaches, we studied the microRNA (miRNA) expression profile during human plasma cell (PC) differentiation induced by stimulation of human blood B cells with T follicular helper cell–dependent signals. Combining the profiles of differentially expressed genes in PC differentiation with gene ontology (GO) analysis revealed that a significant group of genes involved in the transcription factor (TF) activity was preferentially changed. We thus focused on studying the effects of differentially expressed miRNAs on several key TFs in PC differentiation. Cohorts of differentially expressed miRNAs cooperating as miRNA hubs were predicted and validated to modulate key TFs, including a down-regulated miRNA hub containing miR-101-3p, -125b-5p, and -223-3p contributing to induction of *PRDM1* as well as an up-regulated miRNA hub containing miR-34a-5p, -148a-3p, and -183-5p suppressing *BCL6, BACH2*, and *FOXP1*. Induced expression of NF-κB and PRDM1 during PC differentiation controlled the expression of up- and down-regulated miRNA hubs, respectively. Co-expression of miR-101-3p, -125b-5p, and -223-3p in stimulated B cells showed synergistic effects on inhibition of PC formation, which can be rescued by re-introduction of PRDM1. Together, we catalogue the complex roadmap of miRNAs and their functional interplay in collaboratively directing PC differentiation.

Lineage-specific, differentially expressed transcription factors (TFs) and stimulus-induced, stage-specific TFs play pivotal roles in the development and function of B cells. For example, paired box gene 5 (PAX5) controls B-cell identity by instructing B-cell lineage commitment and maintaining mature B-cell function^1^,^2^. B-cell lymphoma 6 (BCL6) is crucial for establishing germinal center (GC) B cells^3^. BTB and CNC homology 2 (BACH2) is required for class-switch recombination in GCs^4^. PR domain zinc finger protein 1 (PRDM1; which is also known as B lymphocyte−induced maturation-1, Blimp-1), X-box binding protein-1 (XBP-1), and interferon regulatory factor 4 (IRF4) are the key TFs that positively control plasma cell (PC) differentiation^5^,^6^,^7^. In particular, PRDM1 is necessary and sufficient for the formation of antibody-secreting PCs^8^. These key TFs form complex gene regulatory circuits that direct B-cell development/differentiation and shape humoral antibody responses following antigen challenges^8^. IRF4 up-regulates *Prdm1*^9^, BCL6 and PRDM1 reciprocally suppress each other^10^,^11^, and BACH2 represses *Prdm1*^12^. PRDM1 suppresses *Pax5*^13^, thereby allowing the de-repression of *Xbp-1*, which is the repressive target of PAX5 and which encodes a protein whose splice variant (XBP-1s) is essential for antibody secretion^6^,^14^. Despite these studies of transcriptional regulation of B-cell development and function, our knowledge of how post-transcriptional regulation by microRNAs (miRNAs) modulates key TFs in controlling the development/differentiation of B cells is limited.

miRNAs finely tune the expression of genes so that their most significant impact on a biological system may occur only when multiple target genes of a miRNA act in a common regulatory pathway or when multiple miRNAs recognize a common target gene^15^,^16^. We thus sought to study the combinatory effects of miRNAs on the regulation of B-cell development/differentiation. In this study, using genome-wide analysis and functional validation of the miRNA expression profile of sorted cell subsets during human PC differentiation *in vitro*, we identified cohorts of miRNAs that are differentially expressed responding to T follicular helper (Tfh) cell-derived signaling. Further computational analysis and experimental verification revealed the co-targeting effects of several differentially expressed miRNAs that jointly formed a regulatory hub to control key TF genes during human PC differentiation.

## Results

### miRNA Expression Profiles during Human PC Differentiation

**Figure 1:**
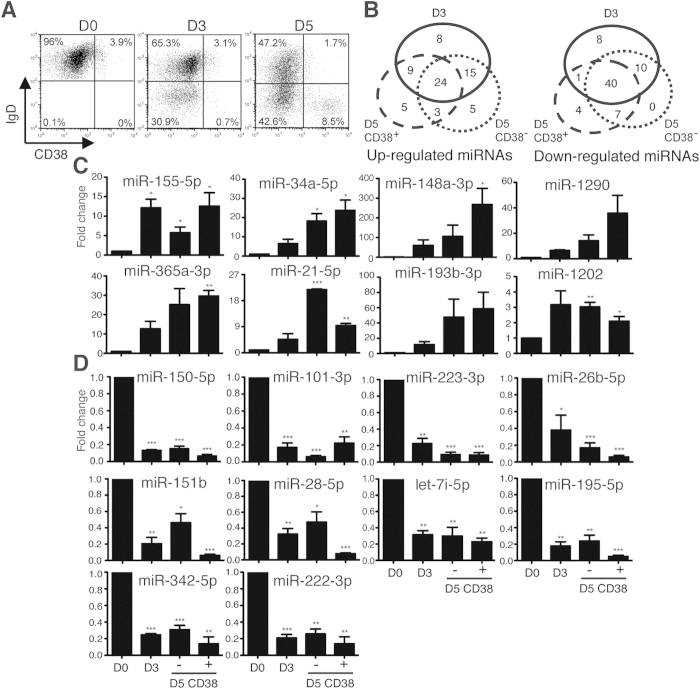
Large-Scale Analysis of miRNA Expression Profiles during Human PC Differentiation. (**A**) Flow cytometric analysis of the expression of IgD and CD38 on isolated human peripheral blood naïve CD27^−^CD19^+^ B cells on day 0 and after treatment with IL-21 and anti-CD40 for 3 and 5 days. (**B**) Number of differentially up-regulated (left) and down-regulated (right) miRNAs in various sorted cell subsets. (**C,D**) RT-QPCR shows the expression of indicated miRNAs in sorted CD38^−^ or CD38^+^ cell subsets that were produced by stimulating naïve B cells with IL-21 + anti-CD40 at day 3 or day 5. miR-155-5p and miR-150-5p serve as the control for up- (**C**) and down-regulated (**D**) miRNAs, respectively. Results represent mean ± SEM (n = 4). *p < 0.05, **p < 0.01, ***p < 0.001.

To study the expression profile of miRNAs that were differentially expressed during the formation of antibody-secreting PCs, we isolated CD27^−^CD19^+^ naïve B cells from healthy donors and treated them with IL-21 and anti-CD40, stimuli that mimic the signals provided by Tfh cells in GCs^17^,^18^. After 3 days in culture, IgD^lo^CD38^hi^ PCs, were not obviously observed; but after 5 days, IgD^lo^CD38^hi^ PCs can be evidently detected (Fig. 1A). Small RNA libraries were then prepared from isolated CD27^−^ naïve B cells at day 0, sorted CD38^−^ cells on days 3 and 5, and sorted CD38^+^ cells on day 5 from two healthy donors for miRNA microarray analysis.

Among the sorted results, 69 and 70 miRNAs were up and down-regulated (>1.5-fold change, p-value <0.05, in both donors), respectively (Table S1), where 24 and 40 ones were up and down-regulated in all subsets (Fig. 1B). In addition, the up/down-regulated miRNAs, even only detected in one subset, mostly had similar expression trend in all subsets (Fig. S1). Dozens of these miRNAs were selected and further validated with RT-QPCR analysis (Fig. 1C,D). The increase in miR-155-5p and the decrease in miR-150-5p were consistent with previous findings in stimulated mouse B cells^19^,^20^,^21^.

Moreover, without sorting, small RNA libraries prepared from day 0 and day 5 cultures of CD19^+^ B cells treated in this manner from other three independent healthy donors were combined (Fig. S2A) and subjected to deep sequencing analysis with the Illumina GA2 platform. A second miRNA microarray was conducted with another small**-**RNA library combined from three other donors. After data processing (see Supplementary Materials and Methods), the expression trend of the unsorted results (Fig. S2B) reconciled the data obtained from sorted subsets (Fig. 1C,D). Some miRNAs that were only represented in one or two experiments may be expressed due to individual variations or the sensitivity of different assay platforms. A panel of miRNAs was selected to confirm their changes in expression from day 0 to day 5 after treatment with IL-21 and anti-CD40 (Fig. S2C). The results with unsorted cells were only used for network construction (Table S2). We suspected the differentially expressed miRNAs that consistently showed up in both sorted and unsorted cells are critical for responding to Tfh cell-derived signals.

### Profiling Gene Expression during Human PC Differentiation

**Figure 2:**
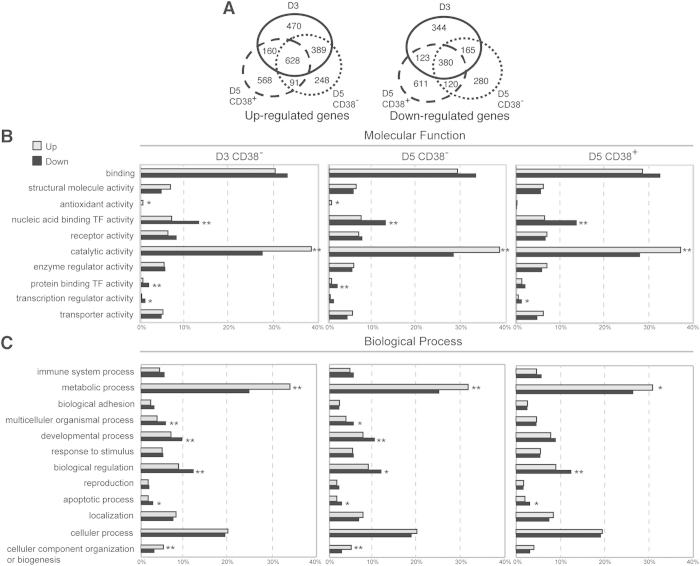
Distribution of Enriched GO Terms of the Up- and Down-Regulated Genes in Various Cell Subsets of Differentiating Human B Cells. (**A**) Number of all up-regulated (left) and down-regulated (right) genes in sorted cell subsets. (**B,C**) The percentage of genes showing significantly up and down-regulated expression during PC differentiation for a given enriched GO term in two major classifications: (**B**) molecular function and (**C**) biological process. The horizontal axis indicates the percentage of individual enriched terms relative to the total enriched terms. Light gray bars and black bars represent the up- and down-regulated genes, respectively. *p < 0.05, **p < 0.01.

To identify the relationship between miRNAs and target genes, we next profile the changes in gene expression during human PC differentiation by using cDNA microarray. The cDNAs derived from three subsets of donor blood B-cell cultures used for miRNA microarray experiments in Fig. 1 were used for microarray analysis of each donor with two replicates. After the within and between sample normalization and data processing (see Supplementary Materials and Methods), we identified 1647, 1356, and 1447 up-regulated genes and 1012, 945 and 1234 down-regulated genes (all p-values <0.05) in day 3 CD38^−^, day 5 CD38^−^ and day 5 CD38^+^ subsets, respectively (Table S3) where 628 and 380 genes were respectively up and down-regulated in all subsets (Fig. 2A).

In addition, we analyzed the functional distribution of all up/down-regulated genes with gene ontology (GO). The enriched GO terms for the up-regulated genes were identified in the molecular functions of catalytic activity (all subsets) and antioxidant activity (day 3 CD38^−^ and day 5 CD38^−^) while the those for down-regulated genes were nucleic acid binding TF activity (all subsets), protein binding TF activity (day 3 CD38^−^ and day 5 CD38^−^), and translation regulator activity (day 3 CD38^−^ and day 5 CD38^+^) (bar-plots in the left of Fig. 2B). Regarding biological process, metabolic process (all cell subsets) and cellular component organization or biogenesis (day 3 CD38^−^ and day 5 CD38^−^) were enriched only for the up-regulated genes, whereas apoptotic process (all subsets), biological regulation (all subsets), developmental process (day 3 CD38^−^ and day 5 CD38^−^), and multicellular organismal process (day 3 CD38^−^ and day 5 CD38^−^) were the selective enriched GO terms for the down-regulated genes (bar-plots in the right of Fig. 2C). However, none of cellular component was enriched in all subpopulations (not shown).

### Putative miRNA Hubs that Target Key TF Genes

**Figure 3:**
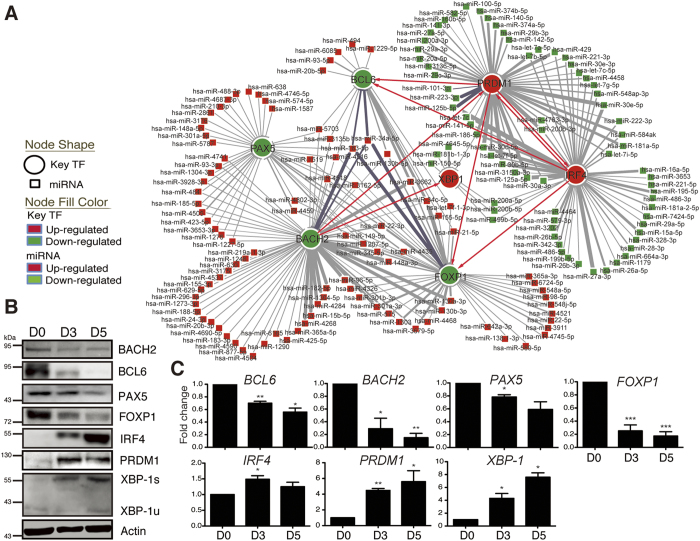
Predicted miRNA-TF Regulatory Networks during Human PC Differentiation. (**A**) Predicted regulatory network showing the differentially expressed miRNAs that putatively target seven key TFs (BCL6, BACH2, FOXP1, PAX5, PRDM1, IRF4, and XBP-1) and the TFs targeted by the others in PC differentiation. The expression of 98 up-regulated and 72 down-regulated miRNAs was negatively correlated with the expression of seven key TFs. (**B,C**) Immunoblot (**B**) and RT-QPCR (**C**) of the expression of key TF proteins during IL-21 + anti-CD40–mediated PC differentiation on the indicated days. Actin was used as the loading control. Results in B are representative of three independent experiments and in (**C**) represent mean ± SEM (n = 3). XBP-1u and XBP-1s are un-spliced and spliced XBP-1, respectively.

Since the molecular functions of differentially expressed genes preferentially act on nucleic acid binding and protein-binding TF activities, we suspected that the differentially expressed miRNAs may contribute to targeting TFs and thus focused on exploring the regulatory relationship between differentially expressed miRNAs and key TF genes during PC differentiation. Several key TFs, particularly four down-regulated TFs (BACH2, BCL6, PAX5, and FOXP1) and three up-regulated TFs (PRDM1, IRF4, and XBP-1), were the chosen potential target genes and a panel of differentially expressed miRNAs identified from sorted and unsorted cells were used. By analyzing with several predicted and validated interactome databases (see Methods), we first predicted the regulatory network between differentially expressed miRNAs and key TFs as well as among these TFs. In several cases, multiple miRNAs were predicted that commonly targeted one key TF gene (Fig. 3A, Table S4). In addition, several miRNAs were commonly connected to more than one key TF. For instance, both miR-183-5p and miR-34a-5p were predicted as commonly targeting *BACH2, BCL6*, and *FOXP1*. We called the miRNAs that commonly targeted to one or more than one TF a miRNA hub and hypothesized that each of these key TF genes is regulated by a miRNA hub consisting of multiple miRNAs. To test this hypothesis, we validated that the changes in protein levels of the key TFs coincided with changes in their mRNA levels (Fig. 3B,C) that also reconciles their reverse association with the differentially expressed miRNA hubs (Fig. 3A).

### Regulation of miRNA Hubs by NF-κB or PRDM1

**Figure 4:**
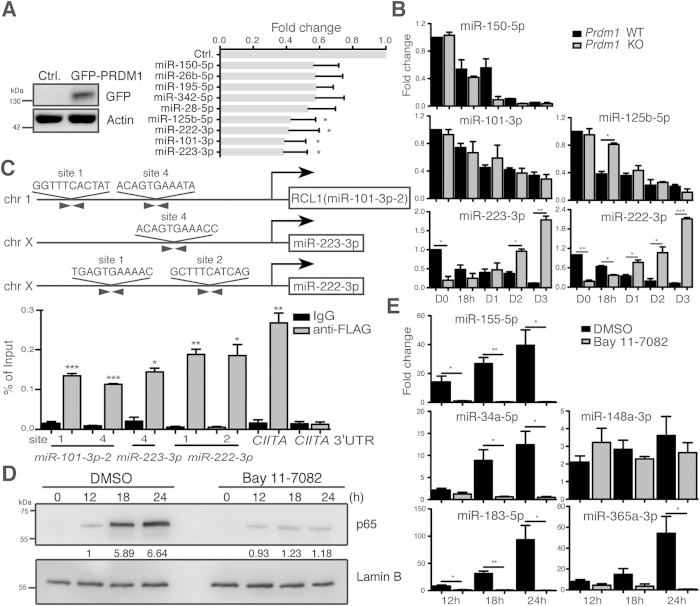
Regulation of miRNAs by NF-κB or PRDM1 during Human PC Differentiation. (**A**) Transduction of a GFP-PRDM1–expressing lentiviral vector reduced the expression of miR-125b-5p, -222-3p, -101-3p, and -223-3p in stimulated human B cells. Immunoblotting and RT-QPCR were performed using sorted GFP^+^ cells from a day 5 culture. (**B**) RT-QPCR shows the expression of the indicated miRNAs after treating *Prdm1* WT or KO splenic B cells with IL-21 + anti-CD40 + anti-IgM for various numbers of days. Data in (**A**,**B**) were normalized to U6. (**C**) PRDM1 bound to *miR-101*-*3p, -222*-*3p*, and *-223*-*3p* loci *in vivo*. Chromatin samples prepared from FLAG-PRDM1-ERD WI-L2 cells induced by CdCl_2_ (5 μM) and 4-hydroxytamoxifen (3 μM) for 24 h were used for ChIP assay with anti-FLAG or control IgG. (**D**) Immunoblot analysis of nuclear lysates of hen egg lysozyme + anti-CD40 + IL-21–stimulated mouse splenic B cells that carry HEL–specific B cell receptors and that were exposed to the NF-κB inhibitor (10 μM) or the solvent control for the indicated time points. Lamin B was used as the loading control. Quantification of immunoblot results is indicated. (**E**) The level of indicated miRNAs in cells treated as in (**D**). Data in (**A**–**C**,**E**) are the mean ± SEM (n = 3). *p < 0.05, **p < 0.01, ***p < 0.001.

We next asked if the identified down-regulated miRNA hubs were suppressed by PRDM1 because PC differentiation is governed by PRDM1. Stimulated human blood B cells were transduced with lentiviral vectors expressing either GFP alone or GFP-tagged PRDM1^22^. The levels of miR-125b-5p, -222-3p, -101-3p, and -223-3p were significantly reduced in human B cells overexpressing PRDM1 (Fig. 4A). Furthermore, we examined if the expression of these miRNAs was perturbed in *Prdm1* knockout (KO) mouse B cells. The levels of miR-150-5p, -125b-5p, -222-3p, and -223-3p were all reduced in anti-IgM + IL-21 + anti-CD40–treated wild type (WT) splenic B cells (Fig. 4B). miR-101-3p, which is not conserved across species, was not reduced (Fig. 4B). Strikingly, in stimulated *Prdm1* KO B cells, the levels of miR-125b-5p were reduced less rapidly at 18 hr; while miR-222-3p and -223-3p were much less reduced than they were in WT cells at later time points (Fig. 4B). Additionally, according to known PRDM1 consensus binding sequences^23^, several PRDM1 binding sites were predicted in the human *miR-101*-*3p, -125b*-*5p, -222*-*3p*, and *-223*-*3p* loci (Fig. S3A). Chromatin immunoprecipitation (ChIP) assay using anti-PRDM1 showed that PRDM1 directly bound to several candidate sites in H929 PC cell line (Fig. S3B). Likewise, induction of PRDM1 in a previously established WI-L2 stable line also led to the binding of PRDM1 to *miR-101*-*3p, -222*-*3p*, and *-223*-*3p* (Fig. 4C, Fig. S3C). *CIITA* promoter III region and its the 3′ untranslated regions (UTR) region were served as the positive and negative control loci for PRDM1 binding, respectively^24^.

Because NF-κB is crucial for inducing GC and antibody production^25^, we examined whether activated NF-κB controls the up-regulated miRNA hub. Indeed, nuclear translocation of the NF-κB subunit p65 in stimulated mouse splenic B cells and the induction of miR-155-5p, -34a-5p, -183-5p, and -365a-3p were all inhibited by treatment with NF-κB inhibitor Bay 11-7082, but miR-148a-3p was not affected (Fig. 4D,E). Together, these data show that induced NF-κB and PRDM1 during PC differentiation activates and suppresses, respectively, these two miRNA hubs.

### *BCL6, BACH2*, and *FOXP1* Are Co-Targeted by miRNA Hubs

**Figure 5:**
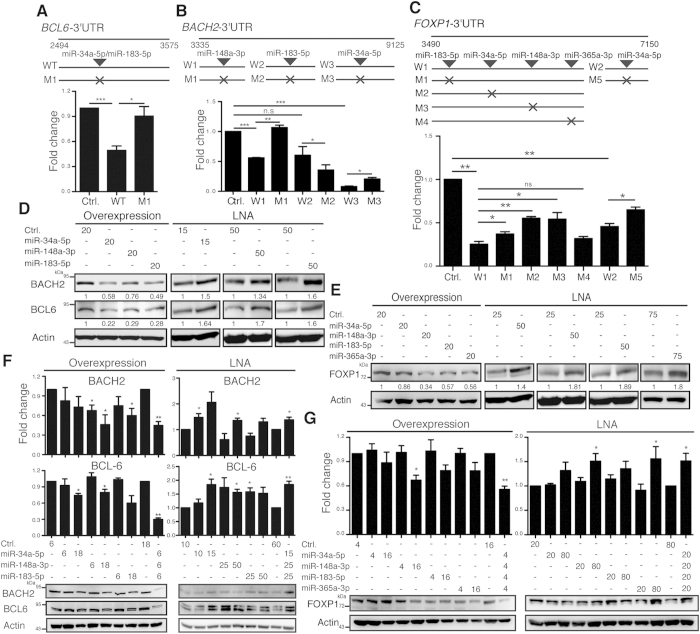
BCL6, BACH2, and FOXP1 Are Repressed by the miR-34a-5p, -148a-3p, -183-5p, -365a-3p Hub. (**A–C**) Relative luciferase activity of the luciferase reporter fused with a 3′UTR region from *BCL6* (**A**), *BACH2* (**B**), or *FOXP1* (**C**) that carried either the wild-type (W) or mutated (M) binding site for the indicated miRNAs. The luciferase assay was performed 24 h after transfection with the indicated plasmids in H929 cells. Results are the mean ± SEM (n = 3). (**D,E**) Immunoblots show the levels of BCL6 and BACH2 (**D**) and FOXP1 (**E**) in SKW6.4 cells transduced with miRNA-expressing lentiviral vectors (moi = 20) or transfected with LNAs 15–75 pmol). Relative band intensities of BCL6, BACH2, and FOXP1 were calculated with densitometric analysis and normalized to the level of the internal control in each corresponding lane. Values were further normalized to control (Ctrl) groups. (**F,G**) Immunoblots of lysates from SKW6.4 cells transduced with lentiviral vectors at the indicated moi or transfected with the indicated amounts (pmol) of LNAs. BCL6 and BACH2 (**F**) and FOXP1 (**G**) protein levels were quantified and normalized as in (**D**,**E**). The bar graph, which shows the mean ± SEM, is the result of at least three independent experiments. *p < 0.05, **p < 0.01, ***p < 0.001, ns = not significant.

To investigate if the identified up-regulated miRNAs directly targets the 3′UTRs of down-regulated TF transcripts, including *BCL6, BACH2*, and *FOXP1*, we generated a series of luciferase reporters that consisted of various fragments of the 3′UTRs of these key TF genes fused to the 3′ end of luciferase cDNA. The predicted miRNA binding sites on the 3′UTRs of key TF genes were individually mutated using site-directed mutagenesis (Fig. 5A–C). Notably, mutating the miR-34a-5p and miR-183-5p cognate sequences in *BCL6* 3′UTR completely de-repressed the luciferase activity (Fig. 5A). Mutating the miR-148a-3p and miR-34a-5p, but not miR-183-5p, binding sites in *BACH2* 3′UTR partially attenuated the repression of luciferase activity (Fig. 5B). Likewise, disruption of the miR-183-5p, miR-34a-5p, or miR-148a-3p site, but not the miR-365a-3p site, attenuated the repression of *FOXP1* 3′UTR–mediated luciferase activity (Fig. 5C).

We then examined if alteration of identified miRNAs influenced the expression of endogenous BCL6, BACH2, and FOXP1. We used both a gain-of-function approach with lentiviral transduction of miRNAs and a loss-of-function approach with transfection with anti-miR locked nucleic acids (LNAs), which are miRNA inhibitors that absorb miRNAs and prevent their interaction with endogenous target transcripts. BCL6 and BACH2 were suppressed by overexpression of miR-34a-5p, -148a-3p, or -183-5p, but their expression was elevated by their corresponding anti-miR LNAs in a lymphoblastoid cell line, SKW6.4, in which mature B cell genes are expressed (Fig. 5D). miR-148a-3p appeared to indirectly regulate endogenous *BCL6* because its conserved binding site was not found in *BCL6* 3′UTR. Overexpression of miR-365a-3p had a similar effect, as did miR-34a-5p, -148a-3p, and -183-5p, on the suppression of endogenous FOXP1 in SKW6.4 cells (Fig. 5E). Conversely, inhibition of these miRNAs with anti-miR LNAs increased endogenous FOXP1 (Fig. 5E).

We next tested if combined alteration of these miRNAs within a miRNA hub could synergize their individual effects on the expression of BCL6, BACH2, or FOXP1. The expression of BACH2 and BCL6 was not changed or was only marginally affected in SKW6.4 cells transduced with lentiviral vectors expressing miR-34a-5p, -148a-3p, or -183-5p at a low multiplicity of infection (moi) (Fig. 5F). However, simultaneous expression of these miRNAs at a low moi induced a more robust reduction of BACH2 and BCL6 than the effects caused by an individual miRNA at a high moi (Fig. 5F). In a reverse trend, transfection with a low dose of individual anti-miR LNAs against miR-34a-5p, -148a-3p, or -183-5p caused minimal effects on the expression of BACH2 and BCL6, but transfection with a mixture of all three anti-miR LNAs at low amounts resulted in substantial increases in the expression of these TFs (Fig. 5F). Similarly, the co-targeting effects of these miRNAs on *FOXP1* were also observed (Fig. 5G). These combined data indicate that miRNAs within a miRNA hub collaboratively inhibit BACH2, BCL6, and FOXP1 during PC differentiation.

**Figure 6:**
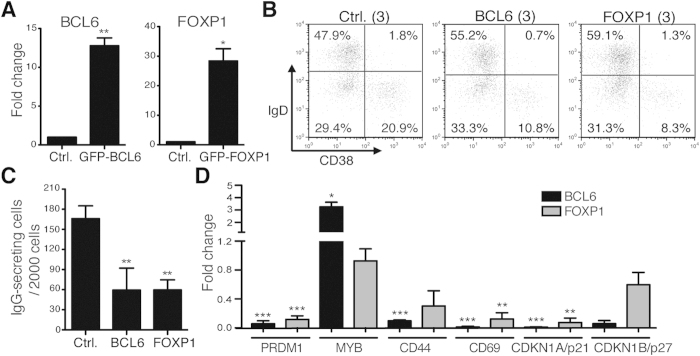
FOXP1 Limits PC Differentiation. (**A**) RT-QPCR analysis of the mRNA levels of BCL6 (left) or FOXP1 (right) by GFP^+^ cells sorted from stimulated human blood B cells transduced with lentiviral vector expressing either GFP-BCL6 (left) or GFP-FOXP1 (right) for 5 days. (**B**) Flow cytometric analysis of surface levels of IgD and CD38 showed that ectopic expression of GFP-FOXP1 and GFP-BCL6 by lentiviral vector (moi = 3) blocked human PC differentiation on day 5 culture. Result is one representative of 3 experiments. (**C**) ELISPOT analysis shows the numbers of IgG-secreting cells by sorted control GFP, GFP-FOXP1 and GFP-BCL6 transduced cells on day 5. (**D**) RT-QPCR shows the expression of indicated genes known to be regulated by BCL6 in GFP-FOXP1 and GFP-BCL6 transduced cells using sorted cells as described in (**A**). Fold change was determined by comparison of the mRNA levels of indicated genes with those in control vector (Ctrl.) transduced cells. Results in (**A**,**C**,**D**) are mean ± SEM (n = 3). *p < 0.05, **p < 0.01, ***p < 0.001.

To understand the role of FOXP1 in human PC differentiation, we transduced a lentiviral vector encoding a GFP-FOXP1 fusion protein into IL-21 + anti-CD40-stimulated human B cells (Fig. 6A) and determined if ectopic expression of FOXP1 affects the formation of PCs. Cells transduced with the GFP-BCL6 expression vector were used as the positive control (Fig. 6A), because Bcl6 negatively controls PC differentiation by suppressing *Prdm1*^11^. Like GFP-BCL6, the expression of GFP-FOXP1 in stimulated human B cells substantially inhibited the formation of IgD^lo^CD38^hi^ PCs compared with cells transduced with the control vector (Fig. 6B). Likewise, similar to those transduced with GFP-BCL6, GFP-FOXP1 expressing cells produced less IgG (Fig. 6C) and failed to up-regulate *PRDM1* mRNA (Fig. 6D). The mRNA levels of B cell activation or cell cycle regulator genes targeted by BCL6, such as *CD44, CD69* and *p21*^10^, are also changed in FOXP1 expressing cells (Fig. 6D). These results suggest that down-regulation of FOXP1, operated by the up-regulated miRNA hub, is required for the formation of PCs.

### Cooperative Effect of miR-101-3p, -125b-5p, and -223-3p on Inhibiting PC Formation

**Figure 7:**
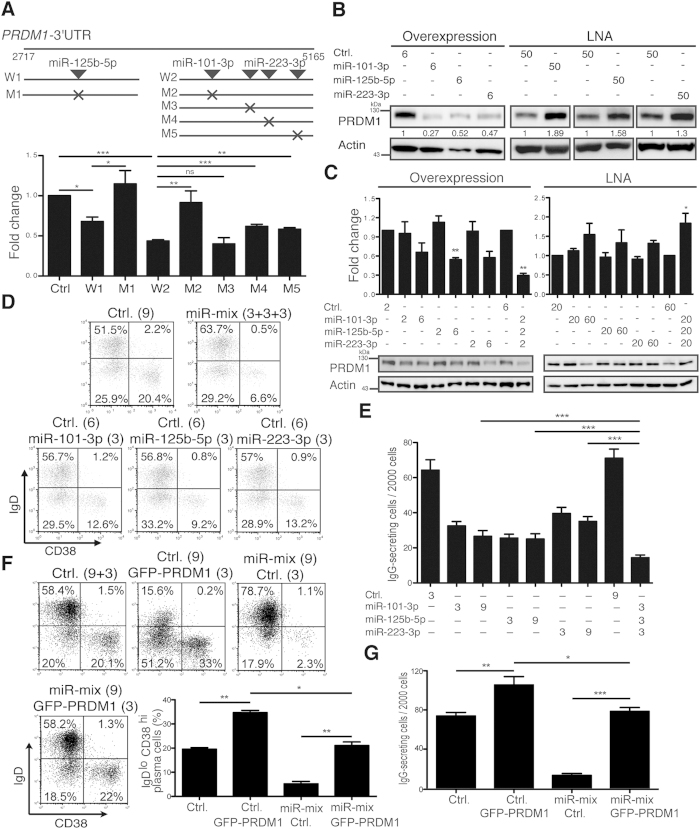
miR-101-3p, -125b-5p, and -223-3p Coordinately Regulate Human PC Differentiation by Targeting *PRDM1*. (**A**) Relative luciferase activity was measured using lysates from 293T cells transfected with luciferase reporters fused with various regions of *PRDM1* WT (W) 3′UTR and mutants (M) carrying the indicated disrupted miRNA binding site. (**B**) H929 cells were transduced with lentiviral vectors expressing the indicated miRNAs (6 moi) or transfected with LNAs (50 pmol), followed by immunoblot analyses. (**C**) Immunoblots of lysates from H929 cells carrying the indicated miRNA (moi) or indicated amounts (pmol) of LNAs. (**D**) Flow cytometric analysis of the levels of IgD and CD38 in the GFP^+^ gate of B cells expressing the indicated miRNAs via lentiviral vectors at day 5. (**E**) ELISPOT analysis shows the number of IgG-secreting out of the total sorted GFP^+^ cells and transduced with control vector, individual miRNAs, or a mixture of miRNAs via expression of lentiviral vectors at day 5. (**F**) Flow cytometric analysis of levels of IgD and CD38 of GFP^+^ cells gated from stimulated human blood B cells and transduced with indicated lentiviral vectors in the day 5 culture. (**G**) ELISPOT analysis shows the number of IgG-secreting cells out of the total GFP^+^ cells sorted from day 5 cultures of cells expressing control GFP (Ctrl.), GFP-PRDM1, a mixture of miRNAs, or miRNAs plus GFP-PRDM1. The moi of each transduction is indicated parenthetically in (**D**,**F**). Results in (**A**,**C**,**E**–**G**) are the mean ± SEM (n = 3, except n = 4 in (**E,G**). *p < 0.05, **p < 0.01, ***p < 0.001, ns = not significant.

We next examined the cooperative effects of the down-regulated miRNAs, including miR-101-3p, -125b-5p, and -223-3p, on human PC differentiation. In the 3′UTR of *PRDM1*, one miR-125b-5p site in the first half of the 3′UTR and one miR-101-3p site and three miR-223-3p sites in the second half of the 3′UTR were predicted by TargetScan (Fig. 7A). As a control^26^,^27^, miR-125b-5p bound to the first half of the 3′UTR of *PRDM1* via conserved cognate sequences because mutating the miR-125b-5p binding site attenuated the repression (Fig. 7A). Disruption of the miR-101-3p site and the second and third, but not the first, miR-223-3p binding sites de-repressed the luciferase activity (Fig. 7A). miR-125b-5p, -101-3p, and -223-3p regulated the expression of PRDM1 because overexpression of either of them decreased PRDM1 protein levels in H929 PCs (Fig. 7B). Blocking the activity of these miRNAs elevated PRDM1 (Fig. 7B). miR-125b-5p, -101-3p, and -223-3p also showed the cooperative effects on PRDM1 expression in contrast to the effects caused by individual miRNAs (Fig. 7C). In a reverse trend, transfection with a low dose of individual anti-miR LNAs against either of them barely affected PRDM1 expression, but transfection with a mixture of all three anti-miR LNAs at low amounts resulted in significant increases in PRDM1 (Fig. 7C).

We then examined if miR-101-3p, -125b-5p, and -223-3p phenotypically influenced human PC differentiation by co-regulating *PRDM1*. Isolated human blood B cells stimulated with IL-21 + anti-CD40 were transduced with either control lentiviral vector or lentiviral vectors producing miR-101-3p, -125b-5p, and/or -223-3p. Simultaneous introduction of miR-101-3p, -125b-5p, and -223-3p exacerbated the inhibitory effects of each miRNAs (Fig. 7D,E). In addition, inhibition of PC formation was linked with down-regulation of *PRDM1* mRNA in all transduced groups (not shown).

To further elucidate if repression of PRDM1 by a mixture of miRNAs attributes to the reduced PC differentiation, we co-transduced another lentiviral vector that encodes GFP alone (Ctrl) or GFP-PRDM1 with or without the vectors producing miR-101-3p, -125b-5p, and -223-3p in stimulated human blood B cells. Flow cytometric analysis of the percentage of IgD^lo^CD38^hi^ PCs in the GFP^+^ gate at day 5 revealed that ectopic expression of GFP-PRDM1 enhanced the generation of IgD^lo^CD38^hi^ PCs as compared with control vector transduced group (Fig. 7F). Ectopic expression of GFP-PRDM1 also promoted the production of IgG (Fig. 7G). More importantly, re-introduction of GFP-PRDM1 that lacks the endogenous *PRDM1* 3′UTR, compensated for the differentiation block caused by a mixture of miRNAs (Fig. 7F,G). Together, these data show that the hub of miR-101-3p, -125b-5p, and -223-3p suppressed PC differentiation largely by inhibiting *PRDM1*.

## Discussion

In this study, we profiled the temporal changes of miRNAs during human PC differentiation using genome-wide approaches. The miRNA expression signatures in various human B-cell subsets obtained from human tonsils, such as naïve B cells, GC B cells, PCs, and memory B cells, have been characterized using miRNA microarrays or expression cloning^28^,^29^. Based on those studies, many miRNAs are selectively expressed at particular stages of B cells. For instance, the level of miR-223 is higher in naïve B cells than in GC B cells^29^. Of note, the levels of several miRNAs including miR-15b, -18b, -93, -148a, -183, -365, -425, and -744 are higher in centroblasts, a type of GC cells experiencing Tfh signals, than in naïve B cells^28^. This observation is consistent with our findings that the levels of these miRNAs were induced in human peripheral B cells treated with Tfh cell-derived stimuli. The differential expression of these miRNAs in centroblasts may reflect their exposure to Tfh stimuli, but the biological significance of these miRNAs in human PC differentiation could not have been assessed before our current study in which we used a primary culture system. Of note, the trend in expression of individual miRNAs within the miRNA hub, such as up-regulation of the miR-34a-5p, -148a-3p, and -183-5p hub and down-regulation of the miR-101-3p, -125b-5p, and -223-3p hub, is similar, supporting that their co-presence or co-absence may have synergistic effects.

When miR-34a-5p, -148a-3p, and -183-5p were individually overexpressed, the fold changes of endogenous BCL-6 protein levels were more dramatic than those of BACH2 and FOXP1, despite that we confirmed that most of these miRNAs can target the 3′UTRs of *BCL6, BACH2*, or *FOXP1*. In particular, the target sites of miR-148a-3p and miR-183-5p in the 3′UTR of *BACH2* are both highly conserved among vertebrates. Therefore, target site conservation is unlikely the major reason, and the efficiency of translation inhibition by miR-34a-5p and -183-5p may contribute to the effects on *BCL6* regulation. We checked the target sites in the 3′UTR and found that only seven sites for miRNA families in vertebrates and three sites for miRNA families in mammals were broadly conserved in the 3′UTR of *BCL6*. In contrast, dozens of conserved sites among vertebrates or only mammals are found in the 3′UTRs of *BACH2* and *FOXP1*. Thus, we speculate that miRNAs targeting a gene transcript with fewer miRNA target sites in the 3′UTR may function more efficiently in translational inhibition than those with more miRNA target sites. In addition, using luciferase reporter assays, we found that miR-183-5p and -365a-3p did not affect *BACH2* and *FOXP1* 3′UTR, respectively, whereas modulation of these miRNAs influenced the expression of BACH2 and FOXP1. Furthermore, some miRNAs may target not only the 3′UTR with a non-seed match^30^ but also the coding regions of mRNAs^31^. Therefore, how individual miRNA works on suppressing the expression of key TFs requires further experiments.

In this study, we found an additional layer of complexity of gene regulatory mechanisms in controlling PC differentiation. Overexpression of miR-101-3p, -125b-5p, or -223-3p individually blocked the formation of PCs, and this inhibitory effect was synergized when they were over-expressed together. Their target genes in this context are not clear, but *PRDM1* appears to be a crucial one because ectopic expression of PRDM1 can rescue the effects of these miRNAs. In addition, miR-223 has been shown to suppress GC marker LIM domain only-2 (*LMO2*) in a B cell lymphoma line^27^, but the roles of miR-223 and LMO2 in PC differentiation are still unknown. As initiation of PC differentiation can occur independently of PRDM1^32^, miRNAs likely play a role in this setting. We here further revealed the mechanisms contributing to the effects of FOXP1 on limiting PC differentiation. *FOXP1* is a target of miR-34a, and knockdown of *FOXP1* in mouse bone marrow progenitor cells phenotypically mimics the effect of miR-34a on the inhibition of early B-cell development^33^. Aberrant expression of FOXP1 results in reduced numbers of GC cells in mice^34^. Additionally, aberrant expression of FOXP1 reduces Ig class-switch recombination to IgG_1_^34^, which reconciled our finding that overexpression of FOXP1 in human peripheral blood B cells reduced the production of IgG. FOXP1 binds to genes associated with cell cycle regulation and proliferation in a diffuse large B-cell lymphoma cell line, OCI-Ly1^34^, and absence of FOXP1 affects cell cycle progression by increasing *p21* and reducing *p27* expression in myocytes^35^. We found that overexpression of *FOXP1* in stimulated B cells not only repressed *PRDM1* mRNA but also reduced *p21* mRNA. Although both BCL6 and FOXP1 can block PC differentiation by reducing the expression of *PRDM1*, we suspect that they may function independently because *p27* is suppressed by BCL6^10^,^11^, but not by FOXP1. Additionally, GC relevant gene *MYB* was induced by BCL6^36^, but not by FOXP1. This notion is supported by the observation that BCL6 and FOXP1 are expressed in distinct spatial locations in human tonsils^34^.

NF-κB is activated by cross-linking CD40 on B cells^37^ and activates miR-155 in transformed B cells^38^. We show that in addition to miR-155-5p, induction of miR-34a-5p, -183-5p, and -365a-3p by IL-21 + anti-CD40 also depends on NF-κB. Furthermore, NF-κB signaling induces *Prdm1*^39^, which in turn switches off the expression of miR-101-3p, -125b-5p, and -223-3p. Together, our results not only illustrate the miRNA/TF regulatory cascades in PC differentiation but also may shed light on new therapies for disorders resulting from dysregulation of PC formation; in particular, miRNAs and decoys consisting of artificial target sites of miRNAs have been proposed as therapeutic approaches^40^,^41^.

## Methods

### Human B cell culture and cell lines

Human blood B cells were isolated from human peripheral blood mononuclear cells of healthy donors obtained from the Taipei Blood Donation Center and were cultured as described^42^. The research involving human subjects was approved by the Institutional Review Board of Academia Sinica and informed consent was obtained from all subjects. The experiments were performed in accordance with the approved guidelines. CD27^−^CD19^+^ and CD19^+^ B cells were purified by RosetteSep (StemCell). Purified human B cells cultured at a density of 1 × 10^6^ cells/mL were stimulated with IL-21 (100 ng/mL; Invitrogen) and anti-CD40 (1 μg/mL; R&D Systems). NCI-H929 (H929) human multiple myeloma cells and SKW6.4 human lymphoblastoid cells were maintained in RPMI 1640 (Life Technologies) containing 10% FBS (Life Technologies), and 2-ME (50 μM; Life Technologies). FLAG-PRDM1-ERD WI-L2 cells were maintained in phenol red-free RPMI 1640 containing 10% C/D FBS (Life Technologies) and Hygromycin B (0.5 mg/mL; invitrogen). All cells were maintained in medium containing penicillin/streptomycin (100 units/mL; Life Technologies).

### Flow cytometry

Cells were harvested and washed with PBS once at a density of 10^6^/mL and the staining procedures were described previously^43^, by using allophycocyanin-conjugated anti-human CD38 (clone HB7), phycoerythrin-conjugated anti-human IgD (clone IA6-2) antibodies. The fluorescence intensity was analyzed by FACSCanto (Becton Dickinson) and FCS Express 3.0 software.

### miRNA experssion profiling using miRNA microarrays and data analysis

Total RNA, isolated by TRI Reagent (Sigma-Aldrich), was used to label with Cy3-pCp fluorescent dyes using the Agilent’s miRNA microarray system V2.0 and hybridized to Agilent human miRNA array (V19) for detecting miRNA expression in unsorted B cells and sorted cell subsets. In brief, total RNA (200 ng) was treated with calf intestine phosphatase for 30 min at 37 °C. 100% DMSO was used for denaturation at 100 °C for 5 min, after which the samples were immediately transferred into an ice water bath for the prevention of reannealing. The samples were then labeled with Cy3-pCp by incubating with T4 RNA ligase for 2 hr at 16 °C. After the labeling reaction, the samples were purified on the Micro Bio-Spin 6 column (Bio-Rad Laboratories, USA). The purified RNA was then denatured and hybridized to the microarray probes at 55 °C, and then spun at 20 rpm for 20 hr in an Agilent Microarray Hybridization Chamber (Agilent Technologies). The microarray slide was subsequently washed and scanned using the Agilent scanner to obtain microarray images. The scanned signals were extracted with Feature Extraction software version 10.7.1.1 using the default setting (Agilent Technologies). In each miRNA array chip, there are 62,344 probes. All array data were done with the between-sample normalization as follow: for each chip, first sorted the probes by their intensities increasingly. Then, for all the chip data, the intensity values of the same sorting order were replaced by the median of the original intensity values at the same order among all chips. In each miRNA chip, expecting for the control ones there are 60,180 probes for 2,006 human miRNAs that each miRNA has 30 probes. For each miRNA, the expressed intensity value was represented by the median of the intensity values of 30 probes. The log2 fold changes of day 3 sample were calculated by the log2 ratios of the expressed intensity value in day 3 over that in day 0. The log2 fold changes of day 5 CD38^+^ and day 5 CD38^−^ subpopulations were also calculated by the same method. Finally, the mean and standard derivation of all fold changes for each normalized array data were calculated. The up and down-regulated miRNAs were defined as their log2 fold changes increasingly over or decreasingly under 1.5 standard derivations in both personal samples (p-value < 0.05).

### TF and miRNA Regulatory Network Construction and Visualization

Seven key TFs, BACH2, BCL6, FOXP1, IRF4, PAX5, PRDM1, and XBP1, were selected to construct their putative regulatory network with differentially expressed miRNAs identified from both sorted and unsorted cell subsets and with each others. The TF-TF interactions were predicted by Ernst *et al.*^44^ and Gerstein *et al.*^45^. The miR-target interactions not only used the results predicted by TargetScanHuman and miRDB, as described above, but also by those from targetHub^46^ (http://app1.bioinformatics.mdanderson.org/tarhub/_design/basic/index.html). targetHub is a database of miRNA-mRNA interactions and the interaction sources include miRTarBase^47^, TargetScan, PicTar4&5^48^, and miRanda^49^. However, the version of miRTarBase was Release 2.5. The latest version of miRTarBase (Release 4.5)^50^ was downloaded and the new interactions related to the seven TFs were added to the interaction data. In addition, TargetScan used in targetHub was version 6.1. The latest version 6.2 was also added for the comparison. These prediction datasets were integrated together, including miRTarBase4.2, TargetScan6.1, TargetScan6.2 conserved and nonconserved, PicTar4, PicTar5, miRanda, and miRDB5.0. In addition, the miRNA expression data for the network construction were used by not only the sorted results but also the unsorted data (Table S2). Finally, the datasets were integrated together and only the pairs that both the miRNAs and target TFs with the reversed regulation were selected. A supporting score for each miR-TF pair was also defined as the total number of how many tools predicted the interaction. All networks were visualized by Cytoscape that was downloaded from (http://www.cytoscape.org/).

### Immunoblotting

Total cell lysates (60 μg) and nuclear lysates (5 μg), prepared as described^42^ or by nuclear extraction kit (Panomics, USA), respectively, were subjected to SDS-PAGE and immunoblotting using primary antibodies against PRDM1^51^, actin (1: 5,000 dilution; Sigma), BACH2 (1: 1,000 dilution; abcam), BCL6 (1: 1,000 dilution; Cell Signaling and 1: 1,000; BioLengend), FOXP1 (1: 1,000 dilution; abcam), PAX5 (1: 500 dilution; Santa Cruz), XBP-1 (1: 500 dilution; Santa Cruz), p65 (1: 500 dilution, Santa Cruz), lamin B (1: 400 dilution; Santa Cruz) and GFP (1: 1,000 dilution; Santa Cruz). The detailed procedures, secondary antibodies used and detection of immunoreactive proteins were as described^42^.

### Luciferase reporter construction and activity assay

3′UTR of *PRDM1, BACH2, BCL6* and *FOXP1*, amplified from the cDNA of primary CD19^+^ peripheral B cells or SKW6.4 cells, was cloned into the downstream of Renilla luciferase cDNA transcribed by the tk promoter (RL-TK). The detailed cloning procedures and the procedures for site-directed mutagenesis of 3′UTR carrying disrupted seed region of miRNA target sites are available upon request. The transfection procedure was performed by electroporation according to a previous reported condition^13^. Briefly, 3 μg of 3′UTR-RL-TK constructs carrying with or without the 3′UTR of various genes were mixed with 0.5 μg of p-GL3-Promoter vector (Promega), as for internal normalization, for electroporation. Twenty-four hours later, electroporated cells were subjected to a dual-luciferase reporter assay as described previously^42^. The fold of repression was calculated as the ratios of Renilla luciferase activities/firefly luciferase to the ratios obtained from control cells transfected with empty vector without carrying 3′UTR of key TFs.

### Lentiviral vector preparation and transduction

The protocol for preparation of lentiviral vectors and viral transduction has been described^43^. Stimulated human B cells, SKW6.4 and H929 cells were transduced with virus at various amounts of moi in the presence of 5 μg/mL polybrene (Sigma-Aldrich). The transduced cells expressing GFP were sorted by FACSAria (Becton Dickinson) one day later for the subsequent experiments.

### RT-quantitative PCR (RT-QPCR)

RNA was isolated by TRI Reagent (Sigma-Aldrich). Small RNA and cDNA synthesis were carried out as described^42^,^52^. The subsequent QPCR analysis on an ABI Prism 7300 sequence detection system (Applied Biosystems) was performed as described^42^. RT-QPCR analysis of miRNAs and mRNAs was carried out using TaqMan probes purchased from ABI. U6 non-coding small nuclear RNA (snRNA) and GAPDH were used as the control for normalization of miRNA and mRNA levels, respectively. PCR was performed for 30 cycles and the primer sequences are described in Table S5.

### Enzyme-linked immunosorbent spot (ELISPOT) assay

ELISPOT analysis for detecting IgG-secreting plasma cells was essentially as described^42^, by using 1 μg of anti-human Ig H+L (SouthernBiotech) coated on each 96-well-plate with PVDF bottom and anti-human IgG (1: 2,000 dilution; SouthernBiotech) as the secondary antibody. Photomicrographs of the spots were analyzed with the AID EliSpot Reader System (AID Autoimmun Diagnostika GmbH).

### Chromatin immunoprecipitation (ChIP)

ChIP assays for analyzing the binding of PRDM1 to endogenous miRNA gene loci were performed essentially as previously described^42^. Briefly, a total of 1 × 10^7^ H929 or WI-L2 cells were used in each anti-PRDM1 or anti-FLAG ChIP assay. Immunoprecipitated chromatin samples were quantified by QPCR using primers that specifically amplified gene fragments encompassing the PRDM1 binding site or within 200 bp of the PRDM1 binding site at each individual miRNA gene locus. Statistical analysis was done by the comparison with the IgG IP samples. Primer sequences used in this study are listed in Table S5. Values obtained from immunoprecipitated samples were normalized to that of their corresponding input samples.

### Statistics

Statistical analysis of experimental data was calculated with a paired, 2-tailed Student’s *t* test. p values lower than 0.05 were considered statistically significant.

## Additional Information

**How to cite this article**: Tsai, D.-Y. *et al.* Uncovering MicroRNA Regulatory Hubs that Modulate Plasma Cell Differentiation. *Sci. Rep.* **5**, 17957; doi: 10.1038/srep17957 (2015).

## Supplementary Material

Supplementary Information

Supplementary Table S1

Supplementary Table S2

Supplementary Table S3

Supplementary Table S4

Supplementary Table S5
