# Supplementary material for: Uncovering MicroRNA Regulatory Hubs that Modulate Plasma Cell Differentiation
Source: Sci Rep. 2015 Dec 11;5:17957. doi: 10.1038/srep17957 (PMC4675970; doi:10.1038/srep17957)
Supplement: Supplementary Information [file srep17957-s1.pdf]

SUPPLEMENTARY INFORMATION

Uncovering MicroRNA Regulatory Hubs that Modulate Plasma Cell Differentiation

Dong-Yan Tsai[1,2], Kuo-Hsuan Hung[1,3], I-Ying Lin[1], Shin-Tang Su[1], Shih-Ying Wu[4,a], Cheng-Han Chung[1,b], Tong-Cheng Wang[1,5], Wen-Hsiung Li[1,5], Arthur Chun-Chieh Shih[4,*], and Kuo-I Lin[1,2,*]

Supplementary Materials and Methods

miRNA deep sequencing and data processing

10 μg of total RNAs, isolated by TRI Reagent (Sigma-Aldrich), from unsorted B cells at day 0 and day 5 were electrophoresed on 15% Urea-TBE-PAGE and smears of miRNA in the 18-30 nt size range were excised. Directional library construction was carried out using the Illumina library preparation kit, by sequential ligation of the 3′- and 5′-adaptor to the miRNA molecules, reverse-transcription into dsDNA, and amplification for 12 cycles by PCR. The reactions were fractionated on 6% TBE-PAGE, and the DNA bands between 92-100 bps were excised. The small RNA libraries were purified and quantified by BioAnalyzer High Sensitivity DNA chip (Agilent, USA). Each library was subjected to one lane of sequencing on Illumina Genome Analyzer II for single-end read of 40 nt. The original raw reads containing any unrecognizable or ambiguous bases were removed. The partial adaptor sequences were trimmed away from the reads if the partial 3'-end sequences are at least 80% similar to partial adaptor sequences (one mismatch allowed when the partial lengths ≤ 8 and two mismatches allowed when the partial lengths > 8). After the trimming process, the trimmed reads of lengths between 16 and 32 nt only were kept and the other reads were filtered out. Then, the miRDeep2 pipeline [1] was used by default parameters to calculate the read counts for each known miRNA. The human genome (hg38, GRCh38) and the miRNA mature and precursor sequences (miRBase Release v21) [2] were downloaded from UCSC Genome Bioinformatics (http://genome.ucsc.edu/) and miRBase (http://www.mirbase.org/), respectively. The internal read mapping tool was Bowtie (version 0.12.8) [3] downloaded from (http://bowtie-bio.sourceforge.net/bowtie2/index.shtml). The miRNAs with < 10 reads in both samples were excluded first. Then, the read counts were normalized by standard quantile normalization using the function implemented in "preprocessCore" package in R. The fold change for each miRNA was calculated by the log2 of the normalized read counts of S2 over S1. We also used DESeq2 [4] to calculate the p-value and adjusted p-values of the fold changes for each miRNA. The over- and under-expressed unsorted miRNAs by deep sequencing were defined as if the read count of the log2 fold change was increased over or decreased under one standard derivation, respectively. However, without replicates, the adjusted p-values were all higher than 0.05. Thus, this deep sequencing result was not used for target gene functional analysis. It was combined with the unsorted miRNA array result together and only as a reference for the key TF and miRNA regulatory network construction. The up- and down-

regulated miRNAs defined in this study were, respectively, at least increased and decreased 1.5 fold-changed in log2 expression ratio following differentiation (Table S2).

Gene expression profiling using cDNA microarrays and data analysis

Total RNA (3 μg) isolated from subpopulations of human peripheral B cells treated with cytokines for 0 and 5 days was prepared by TRI Reagent and used to hybridize with the GeneChip Human Genome U133 Plus 2.0 array. The microarray slides were scanned with a GeneChip scanner 3000 (Affymetrix). The image analysis software was Agilent Feature Extraction 10.5.1.1. Each original CEL file were first processed and normalized by MAS5.0 [5] implemented in R package. For each personal sample, there are four array data sets: day 0, day 3, day 5 $CD38^-$, and day 5 $CD38^+$ subsets, and had done the between-chip normalization as follow: for each data set, the probes were sorted by their intensity values increasingly. Second, the median values of each sorted position were calculated by the averages of the intensity values of each sorted position in all data sets. Third, the values of the same rank in each data set were replaced by the calculated median values. After the within and between-chip normalization, the log2 expression ratios of data from day 3, day 5 $CD38^-$, and day 5 $CD38^+$ subsets were respectively calculated by the log2 of the normalized intensity levels of there samples over that of the sample in day 0. The standard deviations of all log2 expression ratios for each sample were calculated. The up- and down-regulated genes were, respectively, defined that had at least increased and decreased one standard deviation change in log2 expression ratio in all three personal samples following differentiation (p-value < 0.05). One may argue that using one standard derivation may be too loose to get many genes. Here, the standard derivation values in log2 ratio for all cell subsets and donors were all close to 1.8 in average. Therefore, the expression of these genes was increased or decreased almost 4-fold in intensity levels, indicating that their expression levels should have been significantly changed. For unsorted population, the raw data were normalized by the Rank consistent LOWESS method. The p-values were calculated by the distribution of the differential degrees between signals in two samples. Those with low signal/background ratios (cy5/cy3 ratios <1) and high p-values (> 0.01) were removed in our analyses. For each remaining gene, the log2 expression ratio was calculated by the log2 of the normalized intensity levels of samples after treatment over un-treated samples. Similarly, the up- and down-regulated genes were, respectively, defined that had at least increased and decreased 1.5 fold-changed in log2 expression ratio following differentiation.

GO term enrichment analysis

Gene Ontology (GO) categories of up- and down-regulated genes and selected miRNA target genes were analyzed by PANTHER [6]. The PANTHER (protein annotation through evolutionary relationship) classification system (http://www.pantherdb.org/) is a comprehensive system that combines gene function, ontology, pathways and statistical analysis tools. The options of "organism" and "analysis" were selected "*Home sapiens*" and "Functional classification viewed in pie chart", respectively. The p-values of each category by up and down-regulated genes with significant difference were all calculated by Fisher's exact test with a two-tailed mode that was implemented in R.

Mouse B cell culture

Splenic B cells were purified by using anti-B220 microbeads (Miltenyi Biotec) from 7- to 8-week-old C57BL/6, Prdm1$^{f/f}$CD19Cre^{-} (Ctrl), Prdm1$^{f/f}$CD19Cre^{+} (KO) mice [7] and MD4 transgenic mice expressing B cell receptor with specificity for HEL in a C57BL/6 background (purchased from Jackson Laboratory) [8] were then stimulated with IL-21 (200 ng/mL; eBioscience), anti-CD40 (1 μg/mL; BD PharMingen) and anti-IgM (20 μg/mL; Jackson ImmunoReserch Laboratories) or HEL (1 μg/mL; Sigma) in RPMI 1640 containing 10% C/D FBS and penicillin/streptomycin. In one case, stimulated splenic B cells from MD4 transgenic mice were treated with NF-κB inhibitor, Bay 11-7082 (10 μM, Calbiochem). The animal experimental protocol was approved by the Institutional Animal Care and Utilization Committee (IACUC) at Academia Sinica, and the experiments were performed in accordance with the approved guidelines.

Supplementary References

1. Friedlander, M. R., Mackowiak, S. D., Li, N., Chen, W. & Rajewsky, N. miRDeep2 accurately identifies known and hundreds of novel microRNA genes in seven animal clades. *Nucleic Acids Res* **40**, 37-52 (2012).
2. Kozomara, A. & Griffiths-Jones, S. miRBase: annotating high confidence microRNAs using deep sequencing data. *Nucleic Acids Res* **42**, D68-73 (2014).
3. Langmead, B., Trapnell, C., Pop, M. & Salzberg, S. L. Ultrafast and memory-efficient alignment of short DNA sequences to the human genome. *Genome Biol* **10**, R25 (2009).
4. Love, M. I., Huber, W. & Anders, S. Moderated estimation of fold change and dispersion for RNA-seq data with DESeq2. *Genome Biol* **15**, 550 (2014).
5. Pepper, S. D., Saunders, E. K., Edwards, L. E., Wilson, C. L. & Miller, C. J. The utility of MAS5 expression summary and detection call algorithms. *BMC Bioinformatics* **8**, 273 (2007).
6. Mi, H., Muruganujan, A., Casagrande, J. T. & Thomas, P. D. Large-scale gene function analysis with the PANTHER classification system. *Nature protocols* **8**, 1551-1566 (2013).
7. Shapiro-Shelef, M. *et al.* Blimp-1 is required for the formation of immunoglobulin secreting plasma cells and pre-plasma memory B cells. *Immunity* **19**, 607-620 (2003).
8. Goodnow, C. C. *et al.* Altered Immunoglobulin Expression and Functional Silencing of Self-Reactive Lymphocytes-B in Transgenic Mice. *Nature* **334**, 676-682 (1988).

Supplementary Figure Legends

Fig. S1. Large-scale analysis of miRNA expression profiles derived from various B cell subsets.

Clustering heatmap of differentially expressed miRNAs according to different cell subsets derived from $CD19^{+}CD27^{-}$ naïve B cells treated with IL-21 + anti-CD40 for either 3 or 5 days in two independent miRNA array experiments (Array 1 and Array 2).

Fig. S2. Unsorted miRNA expression profiles during human PC differentiation *in vitro.*

(A) Flow cytometric analysis of the expression of IgD and CD38 on isolated human peripheral blood $CD19^{+}$ B cells on day 0 and after treatment with IL-21 and anti-CD40 for 5 days. (B) Clustering heatmap of differentially expressed miRNAs during human PC differentiation *in vitro*. Red represents increased miRNAs, whereas green represents decreased miRNAs based on the presence of their differential expression. (C) RT-QPCR was used to validate the changed levels of 10 up-regulated miRNAs (upper panel) and 10 down-regulated miRNAs (lower panel) between day 0 and day 5 unsorted cultures. All data were normalized to U6 and are presented as the mean ± SEM (n = 3).

Fig. S3. PRDM1 binds directly to several miRNA gene loci.

(A) The graphics show the putative Blimp-1 binding sites and sequences located between −10,000 bp and transcriptional start site, indicated by arrow, of indicated miRNA genes. (B, C) ChIP assay shows the binding of PRDM1 in the indicated site of each miRNA gene. PRDM1 binding to the putative sites in H929 cells was analyzed with ChIP with anti-PRDM1 antibody or an isotype control rabbit IgG (B). PRDM1 binding to the indicated sites of each miRNA gene in FLAG-PRDM1-ERD WI-L2 cells induced by $CdCl_2$ (5 μM) and 4-OHT (3 μM) for 24 h were immunoprecipitated with either anti-FLAG or IgG for a ChIP assay (C). Input DNA was used for normalization in B and C. Results are mean ± SEM (n = 3). *$p < 0.05$, ** $p < 0.01$

Tsai_Sup1.

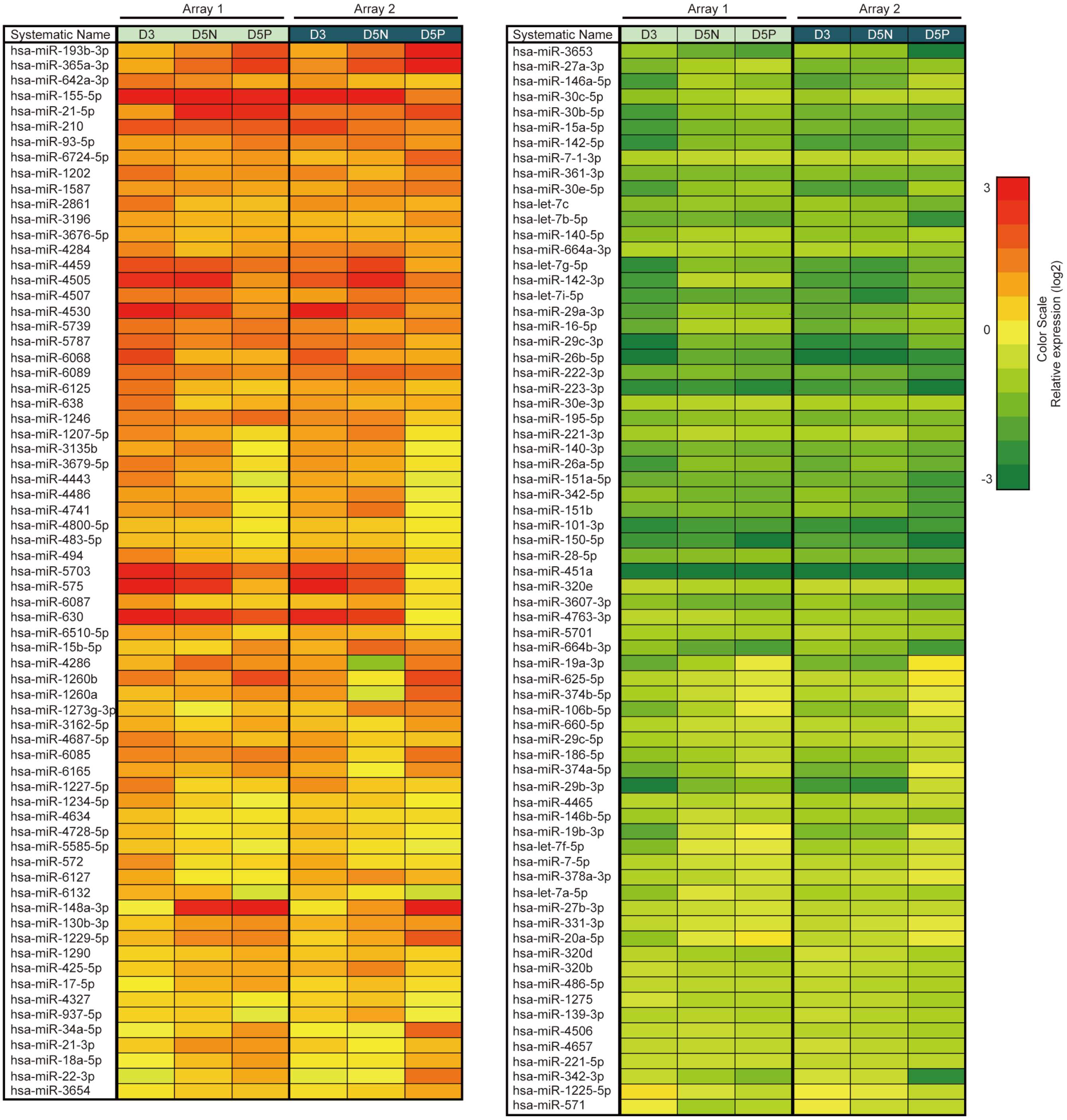
Array 1
Array 2
Systematic Name
D3
D5N
D5P
D3
D5N
D5P
hsa-miR-193b-3p
hsa-miR-365a-3p
hsa-miR-642a-3p
hsa-miR-155-5p
hsa-miR-21-5p
hsa-miR-210
hsa-miR-93-5p
hsa-miR-6724-5p
hsa-miR-1202
hsa-miR-1587
hsa-miR-2861
hsa-miR-3196
hsa-miR-3676-5p
hsa-miR-4284
hsa-miR-4459
hsa-miR-4505
hsa-miR-4507
hsa-miR-4530
hsa-miR-5739
hsa-miR-5787
hsa-miR-6068
hsa-miR-6089
hsa-miR-6125
hsa-miR-638
hsa-miR-1246
hsa-miR-1207-5p
hsa-miR-3135b
hsa-miR-3679-5p
hsa-miR-4443
hsa-miR-4486
hsa-miR-4741
hsa-miR-4800-5p
hsa-miR-483-5p
hsa-miR-494
hsa-miR-5703
hsa-miR-575
hsa-miR-6087
hsa-miR-630
hsa-miR-6510-5p
hsa-miR-15b-5p
hsa-miR-4286
hsa-miR-1260b
hsa-miR-1260a
hsa-miR-1273g-3p
hsa-miR-3162-5p
hsa-miR-4687-5p
hsa-miR-6085
hsa-miR-6165
hsa-miR-1227-5p
hsa-miR-1234-5p
hsa-miR-4634
hsa-miR-4728-5p
hsa-miR-5585-5p
hsa-miR-572
hsa-miR-6127
hsa-miR-6132
hsa-miR-148a-3p
hsa-miR-130b-3p
hsa-miR-1229-5p
hsa-miR-1290
hsa-miR-425-5p
hsa-miR-17-5p
hsa-miR-4327
hsa-miR-937-5p
hsa-miR-34a-5p
hsa-miR-21-3p
hsa-miR-18a-5p
hsa-miR-22-3p
hsa-miR-3654
Array 1
Array 2
Systematic Name
D3
D5N
D5P
D3
D5N
D5P
hsa-miR-3653
hsa-miR-27a-3p
hsa-miR-146a-5p
hsa-miR-30c-5p
hsa-miR-30b-5p
hsa-miR-15a-5p
hsa-miR-142-5p
hsa-miR-7-1-3p
hsa-miR-361-3p
hsa-miR-30e-5p
hsa-let-7c
hsa-let-7b-5p
hsa-miR-140-5p
hsa-miR-664a-3p
hsa-let-7g-5p
hsa-miR-142-3p
hsa-let-7i-5p
hsa-miR-29a-3p
hsa-miR-16-5p
hsa-miR-29c-3p
hsa-miR-26b-5p
hsa-miR-222-3p
hsa-miR-223-3p
hsa-miR-30e-3p
hsa-miR-195-5p
hsa-miR-221-3p
hsa-miR-140-3p
hsa-miR-26a-5p
hsa-miR-151a-5p
hsa-miR-342-5p
hsa-miR-151b
hsa-miR-101-3p
hsa-miR-150-5p
hsa-miR-28-5p
hsa-miR-451a
hsa-miR-320e
hsa-miR-3607-3p
hsa-miR-4763-3p
hsa-miR-5701
hsa-miR-664b-3p
hsa-miR-19a-3p
hsa-miR-625-5p
hsa-miR-374b-5p
hsa-miR-106b-5p
hsa-miR-660-5p
hsa-miR-29c-5p
hsa-miR-186-5p
hsa-miR-374a-5p
hsa-miR-29b-3p
hsa-miR-4465
hsa-miR-146b-5p
hsa-miR-19b-3p
hsa-let-7f-5p
hsa-miR-7-5p
hsa-miR-378a-3p
hsa-let-7a-5p
hsa-miR-27b-3p
hsa-miR-331-3p
hsa-miR-20a-5p
hsa-miR-320d
hsa-miR-320b
hsa-miR-486-5p
hsa-miR-1275
hsa-miR-139-3p
hsa-miR-4506
hsa-miR-4657
hsa-miR-221-5p
hsa-miR-342-3p
hsa-miR-1225-5p
hsa-miR-571
3
0
-3
Color Scale
Relative expression (log2)

Tsai_Sup2.

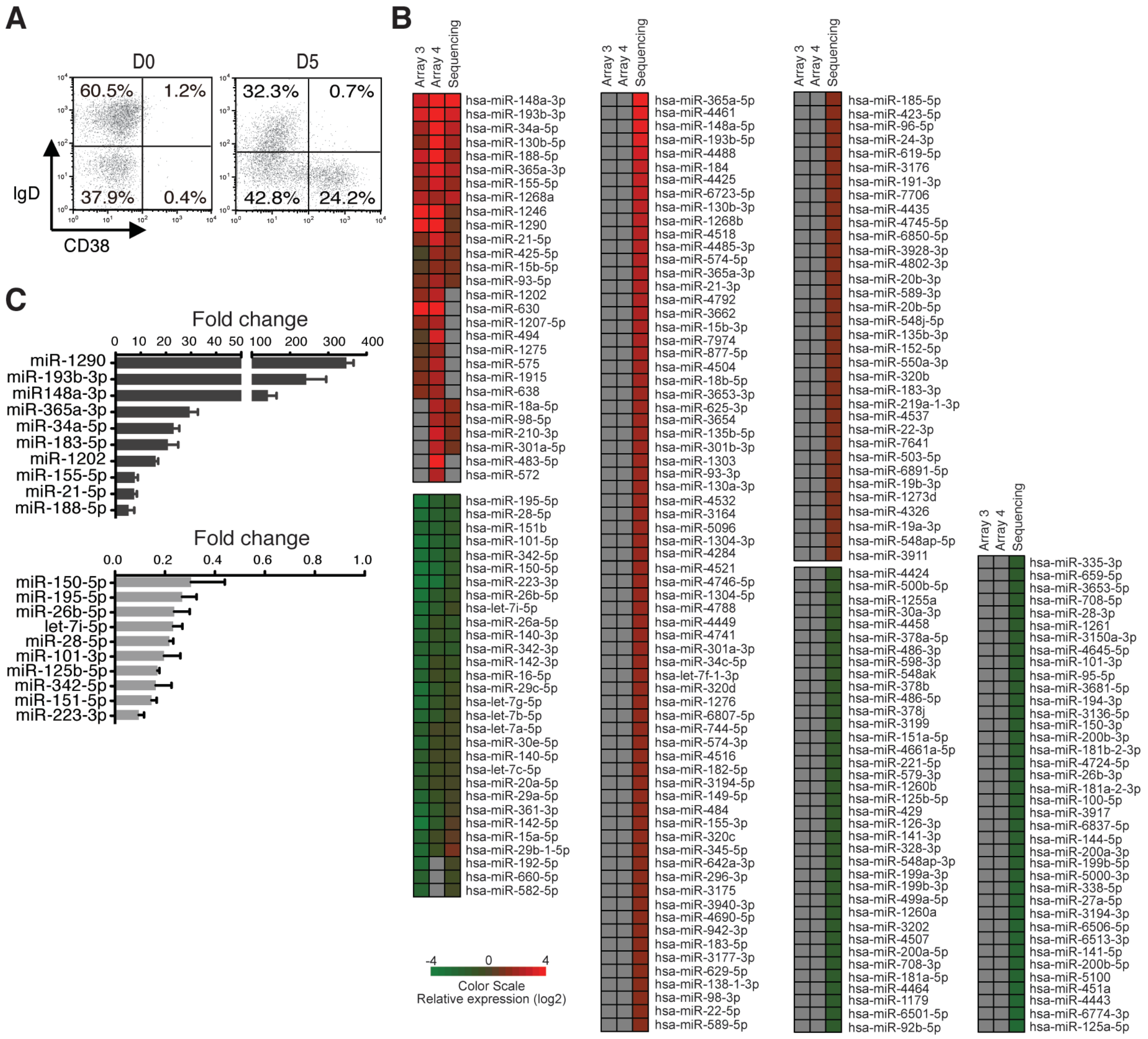

A
D0
60.5%
1.2%
37.9%
0.4%
D5
32.3%
0.7%
42.8%
24.2%
IgD
CD38
B
Array 3
Array 4
Sequencing
hsa-miR-148a-3p
hsa-miR-193b-3p
hsa-miR-34a-5p
hsa-miR-130b-5p
hsa-miR-188-5p
hsa-miR-365a-3p
hsa-miR-155-5p
hsa-miR-1268a
hsa-miR-1246
hsa-miR-1290
hsa-miR-21-5p
hsa-miR-425-5p
hsa-miR-15b-5p
hsa-miR-93-5p
hsa-miR-1202
hsa-miR-630
hsa-miR-1207-5p
hsa-miR-494
hsa-miR-1275
hsa-miR-575
hsa-miR-1915
hsa-miR-638
hsa-miR-18a-5p
hsa-miR-98-5p
hsa-miR-210-3p
hsa-miR-301a-5p
hsa-miR-483-5p
hsa-miR-572
hsa-miR-195-5p
hsa-miR-28-5p
hsa-miR-151b
hsa-miR-101-5p
hsa-miR-342-5p
hsa-miR-150-5p
hsa-miR-223-3p
hsa-miR-26b-5p
hsa-let-7i-5p
hsa-miR-26a-5p
hsa-miR-140-3p
hsa-miR-342-3p
hsa-miR-142-3p
hsa-miR-16-5p
hsa-miR-29c-5p
hsa-let-7g-5p
hsa-let-7b-5p
hsa-let-7a-5p
hsa-miR-30e-5p
hsa-miR-140-5p
hsa-let-7c-5p
hsa-miR-20a-5p
hsa-miR-29a-5p
hsa-miR-361-3p
hsa-miR-142-5p
hsa-miR-15a-5p
hsa-miR-29b-1-5p
hsa-miR-192-5p
hsa-miR-660-5p
hsa-miR-582-5p
-4
0
4
Color Scale
Relative expression (log2)
Array 3
Array 4
Sequencing
hsa-miR-365a-5p
hsa-miR-4461
hsa-miR-148a-5p
hsa-miR-193b-5p
hsa-miR-4488
hsa-miR-184
hsa-miR-4425
hsa-miR-6723-5p
hsa-miR-130b-3p
hsa-miR-1268b
hsa-miR-4518
hsa-miR-4485-3p
hsa-miR-574-5p
hsa-miR-365a-3p
hsa-miR-21-3p
hsa-miR-4792
hsa-miR-3662
hsa-miR-15b-3p
hsa-miR-7974
hsa-miR-877-5p
hsa-miR-4504
hsa-miR-18b-5p
hsa-miR-3653-3p
hsa-miR-625-3p
hsa-miR-3654
hsa-miR-135b-5p
hsa-miR-301b-3p
hsa-miR-1303
hsa-miR-93-3p
hsa-miR-130a-3p
hsa-miR-4532
hsa-miR-3164
hsa-miR-5096
hsa-miR-1304-3p
hsa-miR-4284
hsa-miR-4521
hsa-miR-4746-5p
hsa-miR-1304-5p
hsa-miR-4788
hsa-miR-4449
hsa-miR-4741
hsa-miR-301a-3p
hsa-miR-34c-5p
hsa-let-7f-1-3p
hsa-miR-320d
hsa-miR-1276
hsa-miR-6807-5p
hsa-miR-744-5p
hsa-miR-574-3p
hsa-miR-4516
hsa-miR-182-5p
hsa-miR-3194-5p
hsa-miR-149-5p
hsa-miR-484
hsa-miR-155-3p
hsa-miR-320c
hsa-miR-345-5p
hsa-miR-642a-3p
hsa-miR-296-3p
hsa-miR-3175
hsa-miR-3940-3p
hsa-miR-4690-5p
hsa-miR-942-3p
hsa-miR-183-5p
hsa-miR-3177-3p
hsa-miR-629-5p
hsa-miR-138-1-3p
hsa-miR-98-3p
hsa-miR-22-5p
hsa-miR-589-5p
Array 3
Array 4
Sequencing
hsa-miR-185-5p
hsa-miR-423-5p
hsa-miR-96-5p
hsa-miR-24-3p
hsa-miR-619-5p
hsa-miR-191-3p
hsa-miR-3176
hsa-miR-7706
hsa-miR-4435
hsa-miR-4745-5p
hsa-miR-6850-5p
hsa-miR-3928-3p
hsa-miR-4802-3p
hsa-miR-20b-3p
hsa-miR-589-3p
hsa-miR-20b-5p
hsa-miR-548j-5p
hsa-miR-135b-3p
hsa-miR-152-5p
hsa-miR-550a-3p
hsa-miR-320b
hsa-miR-183-3p
hsa-miR-219a-1-3p
hsa-miR-4537
hsa-miR-22-3p
hsa-miR-7641
hsa-miR-503-5p
hsa-miR-6891-5p
hsa-miR-19b-3p
hsa-miR-1273d
hsa-miR-4326
hsa-miR-19a-3p
hsa-miR-548ap-5p
hsa-miR-3911
hsa-miR-4424
hsa-miR-500b-5p
hsa-miR-1255a
hsa-miR-30a-3p
hsa-miR-4458
hsa-miR-378a-5p
hsa-miR-486-3p
hsa-miR-598-3p
hsa-miR-548ak
hsa-miR-378b
hsa-miR-486-5p
hsa-miR-378j
hsa-miR-3199
hsa-miR-151a-5p
hsa-miR-4661a-5p
hsa-miR-221-5p
hsa-miR-579-3p
hsa-miR-1260b
hsa-miR-125b-5p
hsa-miR-429
hsa-miR-126-3p
hsa-miR-141-3p
hsa-miR-328-3p
hsa-miR-548ap-3p
hsa-miR-199a-3p
hsa-miR-199b-3p
hsa-miR-499a-5p
hsa-miR-1260a
hsa-miR-3202
hsa-miR-4507
hsa-miR-200a-5p
hsa-miR-708-3p
hsa-miR-181a-5p
hsa-miR-4464
hsa-miR-1179
hsa-miR-6501-5p
hsa-miR-92b-5p
Array 3
Array 4
Sequencing
hsa-miR-335-3p
hsa-miR-659-5p
hsa-miR-3653-5p
hsa-miR-708-5p
hsa-miR-28-3p
hsa-miR-1261
hsa-miR-3150a-3p
hsa-miR-4645-5p
hsa-miR-101-3p
hsa-miR-95-5p
hsa-miR-3681-5p
hsa-miR-194-3p
hsa-miR-3136-5p
hsa-miR-150-3p
hsa-miR-200b-3p
hsa-miR-181b-2-3p
hsa-miR-4724-5p
hsa-miR-26b-3p
hsa-miR-181a-2-3p
hsa-miR-100-5p
hsa-miR-3917
hsa-miR-6837-5p
hsa-miR-144-5p
hsa-miR-200a-3p
hsa-miR-199b-5p
hsa-miR-5000-3p
hsa-miR-338-5p
hsa-miR-27a-5p
hsa-miR-3194-3p
hsa-miR-6506-5p
hsa-miR-6513-3p
hsa-miR-141-5p
hsa-miR-200b-5p
hsa-miR-5100
hsa-miR-451a
hsa-miR-4443
hsa-miR-6774-3p
hsa-miR-125a-5p
C
Fold change
0 10 20 30 40 50 100 200 300 400
miR-1290
miR-193b-3p
miR148a-3p
miR-365a-3p
miR-34a-5p
miR-183-5p
miR-1202
miR-155-5p
miR-21-5p
miR-188-5p
Fold change
0.0 0.2 0.4 0.6 0.8 1.0
miR-150-5p
miR-195-5p
miR-26b-5p
let-7i-5p
miR-28-5p
miR-101-3p
miR-125b-5p
miR-342-5p
miR-151-5p
miR-223-3p

Tsai_Sup3.

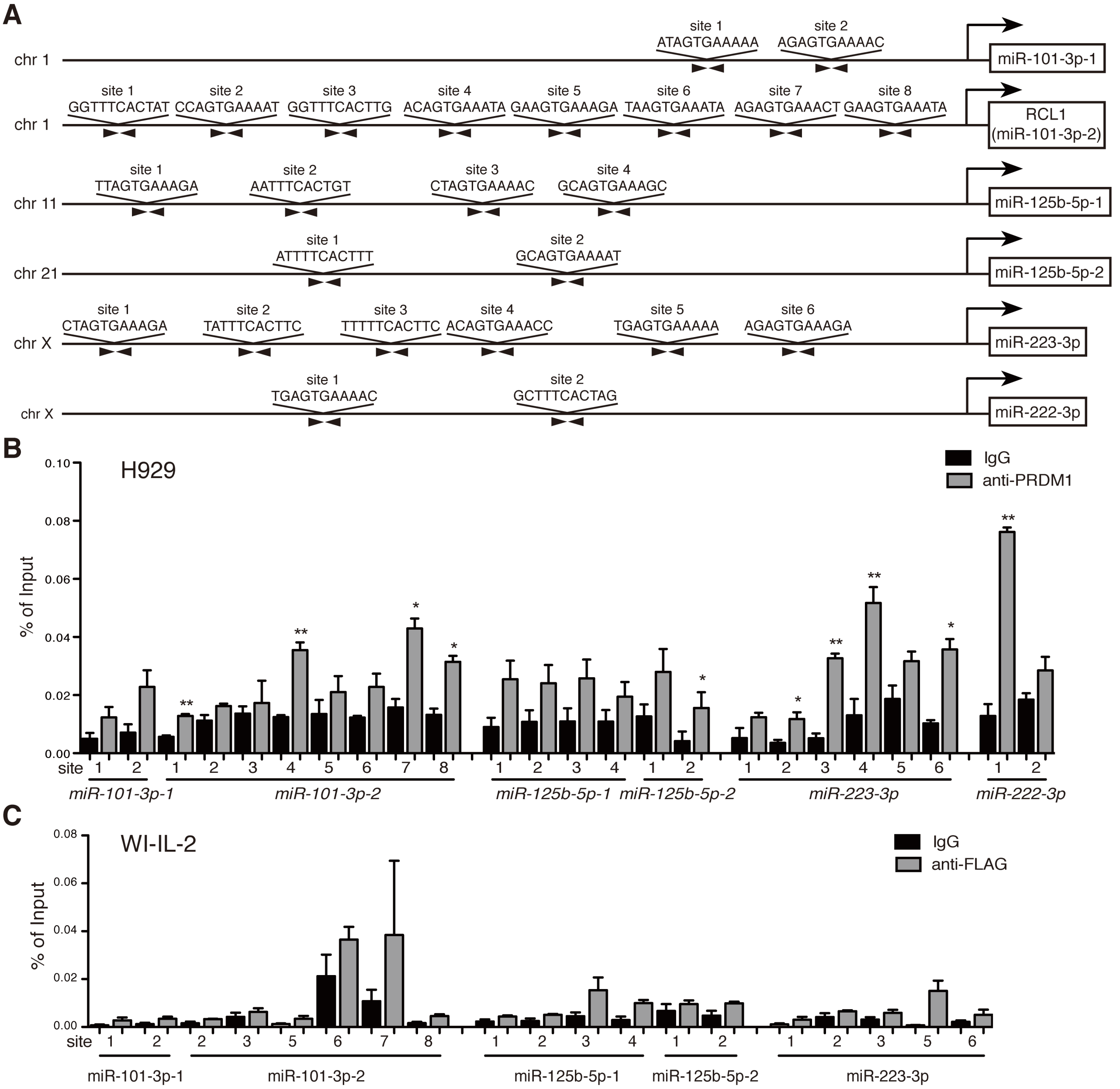
A
chr 1
site 1
ATAGTGAAAAA
site 2
AGAGTGAAAAC
miR-101-3p-1
site 1
GGTTTCACTAT
site 2
CCAGTGAAAAT
site 3
GGTTTCACTTG
site 4
ACAGTGAAATA
site 5
GAAGTGAAAGA
site 6
TAAGTGAAATA
site 7
AGAGTGAAACT
site 8
GAAGTGAAATA
RCL1
(miR-101-3p-2)
chr 11
site 1
TTAGTGAAAGA
site 2
AATTTCACTGT
site 3
CTAGTGAAAAC
site 4
GCAGTGAAAGC
miR-125b-5p-1
chr 21
site 1
ATTTTCACTTT
site 2
GCAGTGAAAAT
miR-125b-5p-2
chr X
site 1
CTAGTGAAAGA
site 2
TATTTCACTTC
site 3
TTTTTCACTTC
site 4
ACAGTGAAACC
site 5
TGAGTGAAAAA
site 6
AGAGTGAAAGA
miR-223-3p
chr X
site 1
TGAGTGAAAAC
site 2
GCTTTCACTAG
miR-222-3p
B
H929
IgG
anti-PRDM1
% of Input
0.10
0.08
0.06
0.04
0.02
0.00
site
miR-101-3p-1
miR-101-3p-2
miR-125b-5p-1
miR-125b-5p-2
miR-223-3p
miR-222-3p
C
WI-IL-2
IgG
anti-FLAG
% of Input
0.08
0.06
0.04
0.02
0.00
site
miR-101-3p-1
miR-101-3p-2
miR-125b-5p-1
miR-125b-5p-2
miR-223-3p
